# Bayesian Regression Analysis for Dependent Data with an Elliptical Shape

**DOI:** 10.3390/e26121072

**Published:** 2024-12-09

**Authors:** Yian Yu, Long Tang, Kang Ren, Zhonglue Chen, Shengdi Chen, Jianqing Shi

**Affiliations:** 1 Department of Statistics and Data Science, College of Science, Southern University of Science and Technology, Shenzhen 518055, China; 12031036@mail.sustech.edu.cn (Y.Y.); 12232884@mail.sustech.edu.cn (L.T.); 2 HUST-GYENNO CNS Intelligent Digital Medicine Technology Center, Wuhan 430074, China; renkang@gyenno.com (K.R.); chenzhonglue@gyenno.com (Z.C.); 3 Department of Neurology and Institute of Neurology, Ruijin Hospital, Shanghai Jiao Tong University School of Medicine, Shanghai 200025, China; chensd@rjh.com.cn; 4 National Center for Applied Mathematics, Shenzhen 518000, China

**Keywords:** ellipse, functional regression model, Gaussian process, nonlinear effects, shape constraints, von Mises–Fisher distribution

## Abstract

This paper proposes a parametric hierarchical model for functional data with an elliptical shape, using a Gaussian process prior to capturing the data dependencies that reflect systematic errors while modeling the underlying curved shape through a von Mises–Fisher distribution. The model definition, Bayesian inference, and MCMC algorithm are discussed. The effectiveness of the model is demonstrated through the reconstruction of curved trajectories using both simulated and real-world examples. The discussion in this paper focuses on two-dimensional problems, but the framework can be extended to higher-dimensional spaces, making it adaptable to a wide range of applications.

## 1. Introduction

Our model is motivated by an analysis of free-living gait data acquired through wearable devices worn by individuals affected by dementia or Parkinson’s disease. Understanding and accurately characterizing gait patterns in these populations is crucial for disease diagnosis and monitoring. We are interested in modeling the trajectories of body center of mass (CoM) excursion during walking. The preprocessed vertical acceleration of the left and right legs is used to study freezing of gait (FoG) detection. One method is to use the phase plot, as shown in [1,2,3], among others. These plots exhibit distinct and diverse oval shapes; see details in Section 4. The aim of this paper is to propose a novel model for trajectory reconstruction with shape constraints. Consequently, the estimated parameters can be used to define new features for disease diagnosis. Similar problems exist in various fields, including biology, transportation, environmental science, and finance. In these domains, the evolution of certain processes is of interest, such as human growth curves [4], mortality and fertility rates [5], traffic flow [6,7], and electricity price curves [8]. These data commonly involve curvilinear relationships or shape constraints. Such constraints, including monotonicity, convexity, or concavity, can be known as a priori or may be required to cope with the model structure. Functional regression with constrained coefficients remains an active topic of research [9,10].

**Figure 2:**
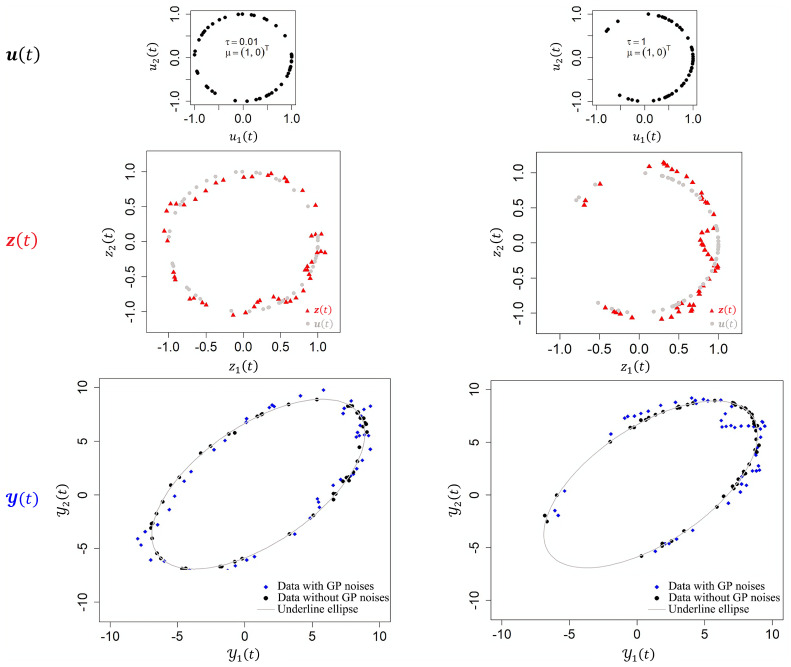
Simulated curves illustrating the hierarchical model structure. (**Top two panels**) The latent variable u(t) follows vMF distribution with distinct parameters. (**Middle two panels**) The latent variable z(t) is governed by a GP. (**Bottom two panels**) Observations y(t) (in blue) alongside the underline values without noises (in black).

The real-world data discussed in Section 4 motivated us to employ ellipse fitting methods. Ellipses are simple and flexible geometric shapes that have attracted considerable attention in areas such as computational metrology [11], augmented reality [12], computer vision [13], computer graphics [14], and pattern recognition [15,16] due to their broad applications [17,18,19,20,21]. Existing ellipse fitting approaches can be broadly categorized into least-squares-based methods [16,22,23,24,25,26,27] and voting or sampling-based methods [28,29]. However, these methods often suffer from ill-conditioned minimization problems, resulting in unstable and suboptimal outcomes, and they can be computationally expensive and memory-intensive [30]. Additionally, these approaches assume that data points lie on a complete ellipse. In this paper, we propose a model to fit curves with shapes of either a complete ellipse or just part of it (see examples in Figure 2), greatly increasing the flexibility of the model and addressing regression problems, making it suitable for a wide range of applications.

Many approaches have been proposed in the literature to capture shape characteristics. Nonparametric methods, such as variational autoencoders using deep neural networks [31,32], often face interpretability issues, reproducibility challenges, and non-identifiability problems. The Bayesian quantile varying coefficient model can flexibly and interpretably capture the nonlinear relationship between the covariates and the response variable [33,34], but they discuss the independent data only. Mixture of Gaussian linear factor models [35,36] can be heavily parameterized and difficult to fit reliably. Alternatively, parametric families of multivariate distributions have been employed to capture skewness and heavy tails in the data [37,38,39], but they may indirectly induce curvature in a complex and difficult-to-interpret manner. Our approach adapts the idea from [40] of a flexible von Mises–Fisher (vMF) distribution, providing clear physical interpretations to functional data with shape constraints.

While we focus on two-dimensional data characterized by an underlying elliptical shape due to their wide range of applications, our framework can be extended to higher-dimensional spheres. To model systematic errors (or individual characteristics) and the dependency of the data, we adopt a nonparametric nonlinear regression model with a Gaussian process (GP) prior. GP regression models are powerful probabilistic models that define distributions over functions, providing a flexible and nonparametric framework for capturing complex patterns and uncertainties in data [41,42,43]. To estimate the model parameters, we employ a Bayesian framework and utilize Markov chain Monte Carlo (MCMC) methods for posterior inference. MCMC allows us to draw samples from the posterior distribution of the model parameters, enabling the calculation of estimates and credible intervals for unknown quantities.

The contributions of this paper are multifaceted. First, our proposed model can fit curves with elliptical shapes. Second, the GP prior allows for the capture of individual characteristics in a single curve when the data contains a set of repeated curves. Thirdly, the estimated parameter values can be utilized to construct new features, resulting in a wide range of applications. The article is organized as follows: Section 2 introduces the ellipse, defines our model structure, and demonstrates how to infer the unknown parameters and latent variables within a Bayesian framework. Some technical details are provided in the Appendix A, Appendix B, Appendix C, Appendix D, Appendix E and Appendix G. Simulation results are presented in Section 3, and two real-world motivating examples for gait analysis are given in Section 4. Section 5 is the discussion.

## 2. Regression Models for Elliptical Shape Curves

### 2.1. The Model

**Figure 3:**
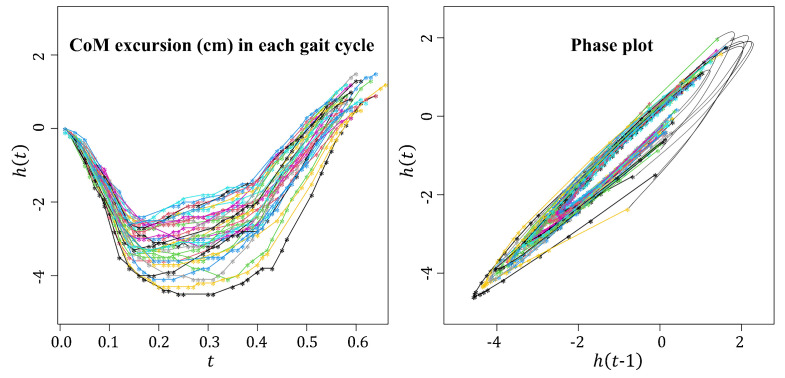
(**Left panel**) Time series representation of CoM (cm) for individual gait cycles, where h(t) denotes the CoM excursion at time *t*. Each line in one color depicts one CoM trajectory during a single gait cycle. (**Right panel**) Corresponding phase plot of CoM excursions. Each point represents the relationship between h(t) and h(t−1).

Let ${\left\{y\left(t_{i}\right)={\left(y_{1}\left(t_{i}\right),y_{2}\left(t_{i}\right)\right)}^{T}\right\}}_{i=1}^{n}$} denote a set of dependent observations collected from an elliptical curve, such as the point locations in the phase plot of the CoM excursion data, as illustrated in Figure 3. Here, $t_{i}$ refers to the time at which the data are recorded. In practice, $t_{i}$ can form a vector of covariates for regression analysis. We propose the following model to fit these data:

(1) $$\begin{array}{cc} y(t_{i}) & =RAz\left(t_{i}\right)+c+\epsilon \left(t_{i}\right),\\ z\left(t_{i}\right)|u\left(t_{i}\right) & ∼\mathrm{GP}\left(u\left(t_{i}\right),K^{*}\left(\cdot ,\cdot ;\theta_{K}\right)\right),\\ u(t_{i}) & \overset{i.i.d.}{∼}\mathrm{vMF}\left(\mu ,\tau \right),\\ \epsilon (t_{i}) & \overset{i.i.d.}{∼}N\left(0,\sigma_{\epsilon }^{2}I_{2}\right),\;i=1,\;\cdots ,\;n. \end{array}$$

Here, $y(t_{i})$ is expressed in terms of a latent variable $z(t_{i})$ subject to rotation (R), stretch (A), and shift (c) transformations. The variable $z(t_{i})$ exhibits smooth variation around a unit circle, which is characterized by a radius of 1 and centered at the origin. We assume a GP prior for $z(t_{i})$, characterized by a mean structure $u(t_{i})$ and a covariance function $K^{*}\left(\cdot ,\cdot ;\theta_{K}\right)$. The mean structure $u(t_{i})$ is located precisely on the unit circle and is assumed to follow a vMF distribution [44,45,46], which will be discussed later. Additionally, the random error $\epsilon (t_{i})$ is assumed to be independent and identically distributed (i.i.d.) Gaussian noise. The rotation (R), stretch (A) and shift (c) along with u(t) of vMF distribution can be treated as a parametric model, while the GP with the covariance function $K^{*}\left(\cdot ,\cdot ;\theta_{K}\right)$ can be treated as a nonparametric model. The parameters involved in the former can be used to define important features (see the details in Section 4.2), while the latter leads to an accurate fitting of the data.

As $z(t_{i})$ varies around the unit circle, its two components, $z_{1}\left(t_{i}\right)$ and $z_{2}\left(t_{i}\right)$, are considered independent and governed by a shared covariance function, denoted as $K(\cdot ,\cdot ;\theta_{K})$. Then, $K^{*}\left(\cdot ,\cdot ;\theta_{K}\right)=I_{2}⊗K\left(\cdot ,\cdot ;\theta_{K}\right)$, where ⊗ denotes the Kronecker product. We adopt $K(\cdot ,\cdot ;\theta_{K})$ as the commonly used square-exponential covariance function, defined as follows:

(2) $$K\left(t,t^{*};\theta_{K}\right)=\nu \exp\left(-\frac{{\left(t-t^{*}\right)}^{2}}{2\omega }\right),\;\theta_{K}=\left(\nu ,\omega \right),$$

where ν>0 and ω>0. Here, ν controls the overall variance, also known as amplitude, and ω is the length scale. In our real-world data examples, *t* denotes the time point. In practice, it could form a vector of covariates, such as a three-dimensional location, to construct the covariance function in a similar manner. The item $∥t-t^{*}∥$ could represent alternative metrics, such as the distance between u(t) and $u(t^{*})$. Other covariance functions can also be utilized; further discussions can be found in [47,48].

**Figure 1:**
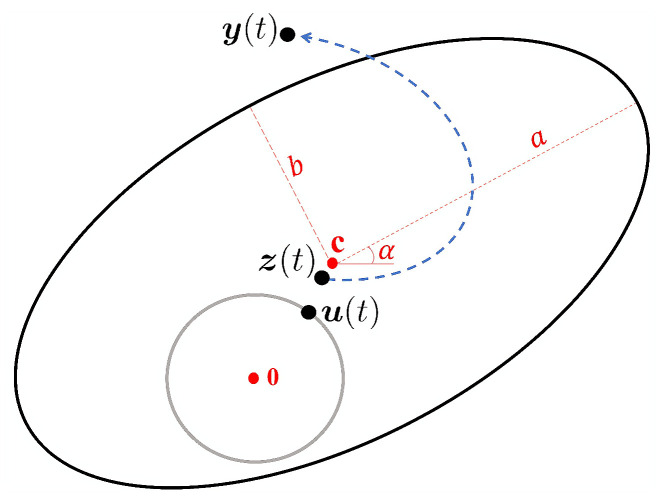
Geometric representation of the transformation from a unit circle to an ellipse. The mean vector u(t) is defined on the unit circle via a von Mises–Fisher (vMF) distribution. The variable z(t) stands for a latent functional variable with mean u(t), exhibiting dependence at different points of *t*. It is transformed into y(t) using Equation (3) to fit the data in an elliptical shape.

An ellipse in a plane can be uniquely described with five parameters: the semi-major axis *a*, the semi-minor axis *b*, the two-dimensional center position $c={\left(c_{1},c_{2}\right)}^{T}$, and the incline angle α. An ellipse can be transformed from a unit circle, as shown in Figure 1. We define a functional variable z(t) using a GP with mean u(t). Here, u(t) is defined on the unit circle via a vMF distribution, and it is transformed using the following formulas:

(3) $$\begin{array}{cc} \; & y(t)=RAz(t)+c, \end{array}$$

(4) $$R=\left(\begin{array}{cc} \cos\alpha & -\sin\alpha \\ \sin\alpha & \cos\alpha \end{array}\right),\;A=\left(\begin{array}{cc} a & 0\\ 0 & b \end{array}\right),$$

where ***R*** is the rotation matrix satisfying $R^{T}R=I$, ***A*** is the stretch matrix, and ***c*** is the shift vector. Therefore, y(t) can be used for fitting data with an elliptical shape.

The vMF distribution is employed to capture the shape structure of the data. This distribution is defined on the (p−1)-sphere in ${ℜ}^{p}$ with the density function given by $p_{p}\left(x;\mu ,\tau \right)=C_{p}\left(\tau \right)\exp\left(\tau \mu^{T}x\right),\;∥x∥=1,$ where $\mu \in {ℜ}^{p}$ represents the mean position (direction), and τ≥0 is the scale parameter that controls the concentration of the distribution around the mean direction μ, as shown in the top two panels of Figure 2. When τ=0, the distribution becomes uniform over the unit circle. The term $C_{p}\left(\tau \right)$ is the normalization constant; a detailed discussion is provided in Appendix A.

Figure 2 demonstrates the simulated model structure for two different cases: one with a nearly uniform distribution of observations (τ=0.01), and another where the observations are clustered within a specific segment (τ=1). The data points generated using the GP exhibit a curvilinear shape, as shown in the bottom two panels, which compare the observed data with and without GP noises.

Let ${\left\{y_{mi}=y_{m}\left(t_{mi}\right),\;i=1,\cdots ,n_{m}\right\}}_{m=1}^{M}$ be repeatedly observed data, where *M* is the number of batches of curves and each curve is independent. Collectively, we denote $Y_{m}={\left(y_{m1},y_{m2},\cdots ,y_{mn_{m}}\right)}^{T}$$\in {ℜ}^{n_{m}\times 2}$ and $Y=\{Y_{1},\cdots ,Y_{M}\}$. Accordingly, we denote $Z_{m}$, ***Z***, $U_{m}$, and ***U***. The notation “→” above a bold capital letter indicates the vectorization of the matrix into a column vector; for example, ${\vec{Y}}_{m}=\mathrm{vec}\left(Y_{m}\right)\in {ℜ}^{2n_{m}\times 1}$. Following the model defined in Equation (1), the observations have the following distributions:

(5) $$\begin{array}{cc} \left({\vec{Y}}_{m}|{\vec{Z}}_{m},a,b,c,\alpha ,\sigma_{\epsilon }^{2}\right) & ∼N\left(\left[RA⊗I_{n_{m}}\right]{\vec{Z}}_{m}+c⊗1_{n_{m}}\;,\;\sigma_{\epsilon }^{2}I_{2n_{m}}\right),\\ \left({\vec{Z}}_{m}|{\vec{U}}_{m},\theta_{K}\right) & ∼N\left({\vec{U}}_{m}\;,\;I_{2}⊗K_{m}\right),\\ \left({\vec{U}}_{m}|\tau ,\mu \right) & ∼{\mathrm{vMF}}_{n_{m}}\left(\mu ,\;\tau \right), \end{array}$$

where $K_{m}\in {ℜ}^{n_{m}\times n_{m}}$, and the (i,j)-th entry of $K_{m}$ is denoted as $K_{mij}=K\left(t_{mi},t_{mj};\theta_{K}\right)$, calculated using Equation (2). The probability density function for ${\vec{U}}_{m}$ is

$$p\left({\vec{U}}_{m}|\tau ,\mu \right)={\left(2\pi I_{0}\left(\tau \right)\right)}^{-n}\exp\left({\left[\tau \mu ⊗1_{n}\right]}^{T}{\vec{U}}_{m}\right).$$

In practice, we may also employ the following simplified model:

$$\begin{array}{cc} y\left(t_{i}\right)|z\left(t_{i}\right),u\left(t_{i}\right) & =RAu\left(t_{i}\right)+z\left(t_{i}\right)+c+\epsilon \left(t_{i}\right),\\ z(t_{i}) & ∼\mathrm{GP}\left(0,K^{*}\left(\cdot ,\cdot ;\theta_{K}\right)\right),\\ u(t_{i}) & \overset{i.i.d.}{∼}\mathrm{vMF}\left(\mu ,\tau \right),\\ \epsilon (t_{i}) & \overset{i.i.d.}{∼}N\left(0,\sigma_{\epsilon }^{2}I_{2}\right),\;i=1,\;\cdots ,\;n, \end{array}$$

which is a simpler version of (1). The main distinction is the separation of the GP from the parameters of the ellipse, therefore simplifying the estimation process. However, the covariance and cross-covariance functions for $z(t_{i})$ must be considered with care, as the assumption of independence between the two components of $y(t_{i})$ may not be appropriate for data distributed around an ellipse. The Bayesian inference framework discussed in the following section for model (1) can be readily extended to this model without difficulties.

### 2.2. Bayesian Inference

The MCMC algorithm is employed to obtain posterior distributions and facilitate probabilistic inferences about the unknown variables and parameters based on observed data. The primary quantities of interest in the model include the latent variables $\vec{Z}={\left\{{\vec{Z}}_{m}\right\}}_{m=1}^{M}$, latent variables $\vec{U}={\left\{{\vec{U}}_{m}\right\}}_{m=1}^{M}$, and parameters defined as: $\Theta =\left\{a,b,c,\alpha ,\tau ,\mu ,\theta_{K},\sigma_{\epsilon }^{2}\right\}$.

#### 2.2.1. Sampling Algorithm

A Gibbs sampler [49,50] is utilized to iteratively generate random values for the parameters and latent variables. The (s+1)-th iteration consists of the following steps:

Step 1: Generate ${\vec{Z}}^{\left(s+1\right)}∼p\left(\vec{Z}|\vec{Y},{\vec{U}}^{\left(s\right)},\Theta^{\left(s\right)}\right)$. As $p\left(\vec{Z}|\vec{Y},{\vec{U}}^{\left(s\right)},\Theta^{\left(s\right)}\right)=\prod_{m=1}^{M}p\left({\vec{Z}}_{m}|{\vec{Y}}_{m},{{\vec{U}}_{m}}^{\left(s\right)},\Theta^{\left(s\right)}\right).$ This can be decomposed into generating each ${{\vec{Z}}_{m}}^{\left(s+1\right)}∼p\left({\vec{Z}}_{m}|{\vec{Y}}_{m},{{\vec{U}}_{m}}^{\left(s\right)},\Theta^{\left(s\right)}\right)$ separately for m=1,⋯,M.
Step 2: Generate ${\vec{U}}^{\left(s+1\right)}∼p\left(\vec{U}|\vec{Y},{\vec{Z}}^{\left(s+1\right)},\Theta^{\left(s\right)}\right)$. As $p\left(\vec{U}|\vec{Y},{\vec{Z}}^{\left(s+1\right)},\Theta^{\left(s\right)}\right)=\prod_{m=1}^{M}p\left({\vec{U}}_{m}|{\vec{Y}}_{m},{{\vec{Z}}_{m}}^{\left(s+1\right)},\Theta^{\left(s\right)}\right).$ This can be decomposed into generating each ${{\vec{U}}_{m}}^{\left(s+1\right)}∼p\left({\vec{U}}_{m}|{\vec{Y}}_{m},{{\vec{Z}}_{m}}^{\left(s+1\right)},\Theta^{\left(s\right)}\right)$ separately for m=1,⋯,M.
Step 3: Generate $\Theta^{\left(s+1\right)}∼p\left(\Theta |\vec{Y},{\vec{Z}}^{\left(s+1\right)},{\vec{U}}^{\left(s+1\right)}\right)$ based on predefined hyperpriors for each parameter using the Metropolis-Hasting algorithm.

The sampling procedure, conditional distributions, and specified hyperpriors involved in the above steps are detailed in the following for each respective step. For clarity, the superscripts have been omitted.

#### 2.2.2. Generate $\vec{Z}∼p\left(\vec{Z}|\vec{Y},\vec{U},\Theta \right)$

In Step 1, the distribution of ${\vec{Z}}_{m}$ given ${\vec{Y}}_{m}$, ${\vec{U}}_{m}$, and all other parameters is proportional to the product of two Gaussian distributions:

$$\begin{array}{cc} \; & p\left({\vec{Z}}_{m}|{\vec{Y}}_{m},{\vec{U}}_{m},\Theta \right)\\ \propto & p\left({\vec{Y}}_{m}|{\vec{Z}}_{m},{\vec{U}}_{m},\Theta \right)\cdot p\left({\vec{Z}}_{m}|{\vec{U}}_{m},\Theta \right)\\ = & p\left({\vec{Y}}_{m}|{\vec{Z}}_{m},\Theta \right)\cdot p\left({\vec{Z}}_{m}|{\vec{U}}_{m},\Theta \right)\\ \propto & \exp\{-\frac{1}{2}{\left({\vec{Z}}_{m}-\left[A^{-1}R^{T}⊗I_{n_{m}}\right]\left[{\vec{Y}}_{m}-c⊗1_{n_{m}}\right]\right)}^{T}\left[\frac{1}{\sigma_{\epsilon }^{2}}A^{2}⊗I_{n_{m}}\right]\\ \; & \;\left({\vec{Z}}_{m}-\left[A^{-1}R^{T}⊗I_{n_{m}}\right]\left[{\vec{Y}}_{m}-c⊗1_{n_{m}}\right]\right)-\frac{1}{2}{\left({\vec{Z}}_{m}-{\vec{U}}_{m}\right)}^{T}\left[I_{2}⊗K_{m}^{-1}\right]\left({\vec{Z}}_{m}-{\vec{U}}_{m}\right)\}. \end{array}$$

Based on the properties from the multivariate Gaussian distribution, the conditional distribution of ${\vec{Z}}_{m}$ remains a multivariate Gaussian distribution. The derivation is provided in Appendix B:

$$\begin{array}{cc} \left({\vec{Z}}_{m}|{\vec{Y}}_{m},{\vec{U}}_{m},\Theta \right) & ∼N\left(\Sigma_{zm}\left(\frac{1}{\sigma_{\epsilon }^{2}}\left[AR^{T}⊗I_{n_{m}}\right]\left[{\vec{Y}}_{m}-c⊗1_{n_{m}}\right]+\left[I_{2}⊗K_{m}^{-1}\right]{\vec{U}}_{m}\right),\;\Sigma_{zm}\right),\\ \Sigma_{zm} & =\sigma_{\epsilon }^{2}\left[A^{-2}⊗I_{n_{m}}\right]\left[I_{2}⊗K_{m}\right]{\left(\sigma_{\epsilon }^{2}\left[A^{-2}⊗I_{n_{m}}\right]+I_{2}⊗K_{m}\right)}^{-1}\\ \; & =\left[I_{2}⊗K_{m}\right]{\left(I_{2n_{m}}+\sigma_{\epsilon }^{-2}\left[A^{2}⊗K_{m}\right]\right)}^{-1}. \end{array}$$

Despite the availability of analytical forms for the conditional distribution, directly working with or evaluating the density becomes computationally burdensome when the number of data points per batch $n_{m}$ is large, as it involves solving the inverse of the matrix $\left(\sigma_{\epsilon }^{2}\left[A^{-2}⊗I_{n_{m}}\right]+I_{2}⊗K_{m}\right)$. To avoid these computational costs, some efficient algorithms are recommended, such as nearest neighbor GP [51,52,53,54,55], along with other methods [56,57,58,59,60].

#### 2.2.3. Generate $\vec{U}∼p\left(\vec{U}|\vec{Y},\vec{Z},\Theta \right)$

In Step 2, the distribution of ${\vec{U}}_{m}$ given ${\vec{Y}}_{m}$, ${\vec{Z}}_{m}$, and all other parameters is proportional to the product of a Gaussian distribution and a multivariate vMF distribution:

(6) $$\begin{array}{cc} \; & p\left({\vec{U}}_{m}|{\vec{Y}}_{m},{\vec{Z}}_{m},\Theta \right)\\ = & p\left({\vec{U}}_{m}|{\vec{Z}}_{m},\Theta \right)\\ \propto & p\left({\vec{Z}}_{m}|{\vec{U}}_{m},\Theta \right)\cdot p\left({\vec{U}}_{m}|\Theta \right)\\ \propto & \exp\left\{\left(\tau \mu^{T}⊗{1_{n_{m}}}^{T}+{{\vec{Z}}_{m}}^{T}\left[I_{2}⊗K_{m}^{-1}\right]\right){\vec{U}}_{m}-\frac{1}{2}{{\vec{U}}_{m}}^{T}\left[I_{2}⊗K_{m}^{-1}\right]{\vec{U}}_{m}\right\}. \end{array}$$

The conditional distribution of each $u_{mi}$ follows the Fisher-Bingham (FB) distribution [61]:

$$\begin{array}{cc} \left(u_{mi}|{\vec{Z}}_{m},\Theta \right)∼\; & \mathrm{FB}\left(\tau \mu +\delta^{-1}z_{mi},\;\frac{1}{2\delta }I_{2}\right),\;i=1,\cdots ,n_{m}, \end{array}$$

where $\delta =K_{m[i,i]}-K_{m[i,-i]}{\left(K_{m[-i,-i]}\right)}^{-1}{\left(K_{m[i,-i]}\right)}^{T}$, $K_{m[i,i]}$ is the (i,i)-th entry of $K_{m}$, $K_{m[i,-i]}$ represents the *i*-th row vector from $K_{m}$ excluding the *i*-th item, and $K_{m[-i,-i]}$ denotes the submatrix of $K_{m}$ excluding the *i*-th row and *i*-th column. The density for the FB distribution is detailed in Appendix A, and the proof for this posterior conditional distribution is given in Appendix C.

However, generating random samples from the FB distribution is intractable. The difficulty is that the normalized constant is hard to be calculated analytically, the computation necessitates to be approximated with numerical methods [62,63,64]. Our simulation approach uses the first-ever general-purpose acceptance/rejection simulation algorithm proposed in [65], implemented with the “simdd” package in R, which is highly efficient and user-friendly. Ref. [66] gives a Las Vegas polynomial time algorithm for sampling from the Bingham distribution based on rejection sampling; however, the addressing of the calculations relevant to the FB distribution when a linear term is added remains an open problem, as noted in their discussion.

Despite this, the FB method requires generating ${\left\{u_{mi}\right\}}_{i=1}^{n_{m}}$ one by one, which is time-consuming when the number of data points $n_{m}$ in each batch is large. In our study, we also consider a Gaussian approximation (GA) method to address this issue. The GA method, also known as the Laplace approximation, is discussed in [67,68]. First, using the density Equation (6), we can derive the mode estimation for ${\vec{U}}_{m}$ as follows:

(7) $$\begin{array}{cc} \log p\left({\vec{Z}}_{m},{\vec{U}}_{m}|\Theta \right) & =\log p\left({\vec{Z}}_{m}|{\vec{U}}_{m},\Theta \right)+\log p\left({\vec{U}}_{m}|\Theta \right)\\ \; & \propto -\frac{1}{2}{\left({\vec{Z}}_{m}-{\vec{U}}_{m}\right)}^{T}\left[I_{2}⊗K_{m}^{-1}\right]\left({\vec{Z}}_{m}-{\vec{U}}_{m}\right)+{\left[\tau \mu ⊗1_{n_{m}}\right]}^{T}{\vec{U}}_{m}. \end{array}$$

Taking the first derivative with respect to ${\vec{U}}_{m}$ and letting ${\left.\frac{\partial \log p\left({\vec{Z}}_{m},{\vec{U}}_{m}|\Theta \right)}{\partial {\vec{U}}_{m}}\right|}_{{\vec{U}}_{m}={\hat{\vec{U}}}_{m}}=0$, we have

$${\hat{\vec{U}}}_{m}=\left[I_{2}⊗K_{m}\right]\left[\tau \mu ⊗1_{n_{m}}\right]+{\vec{Z}}_{m}.$$

Next, we approximate $\log p\left({\vec{Z}}_{m}|{\vec{U}}_{m},\Theta \right)$ by Taylor expansion to the second order,

(8) $$\begin{array}{cc} \; & \log p\left({\vec{Z}}_{m}|{\vec{U}}_{m},\Theta \right)\\ \propto & -\frac{1}{2}{\left({\vec{Z}}_{m}-{\vec{U}}_{m}\right)}^{T}\left[I_{2}⊗K_{m}^{-1}\right]\left({\vec{Z}}_{m}-{\vec{U}}_{m}\right)\\ \approx & \log p\left({\vec{Z}}_{m}|{\hat{\vec{U}}}_{m},\Theta \right)+{\left({\left.{\frac{\partial \log p\left({\vec{Z}}_{m}|{\vec{U}}_{m},\Theta \right)}{\partial \vec{U}\;\;}}_{m}\right|}_{{\vec{U}}_{m}={\hat{\vec{U}}}_{m}}\right)}^{T}{\vec{U}}_{m}\\ \; & \;+\frac{1}{2}{{\vec{U}}_{m}}^{T}{\left({\left.\frac{\partial^{2}\log p\left({\vec{Z}}_{m}|{\vec{U}}_{m},\Theta \right)}{\partial {\vec{U}}_{m}\partial {{\vec{U}}_{m}}^{T}}\right|}_{{\vec{U}}_{m}={\hat{\vec{U}}}_{m}}\right)}^{T}{\vec{U}}_{m}\\ \approx & -\frac{1}{2}{\left[\tau \mu ⊗1_{n_{m}}\right]}^{T}\left[I_{2}⊗K_{m}\right]\left[\tau \mu ⊗1_{n_{m}}\right]-{\left[\tau \mu ⊗1_{n_{m}}\right]}^{T}{\vec{U}}_{m}-\frac{1}{2}{{\vec{U}}_{m}}^{T}\left[I_{2}⊗K_{m}^{-1}\right]{\vec{U}}_{m}. \end{array}$$

By substituting the result from Equation (8) into (7), since $p\left({{\vec{U}}_{m}|\vec{Z}}_{m},\Theta \right)\propto p\left({\vec{Z}}_{m},{\vec{U}}_{m}|\Theta \right)$, we have

$$\log p\left({\vec{U}}_{m}|{\vec{Z}}_{m},\Theta \right)\propto -\frac{1}{2}{\left({\vec{U}}_{m}-{\hat{\vec{U}}}_{m}\right)}^{T}\left[I_{2}⊗K_{m}^{-1}\right]\left({\vec{U}}_{m}-{\hat{\vec{U}}}_{m}\right).$$

Thus, the GA for $\left({\vec{U}}_{m}|{\vec{Z}}_{m},\Theta \right)$ is given by $N\left({\hat{\vec{U}}}_{m}\;,\;I_{2}⊗K_{m}\right)$. However, samples generated by the GA method violate the norm-1 constraint; therefore, the paired points ${\left\{u_{mi}\right\}}_{i=1}^{n_{m}}$ in the generated ${\vec{U}}_{m}$ will be normalized to ensure they lie on the unit circle.

The comparison between the GA method and the FB approach for sampling ${\vec{U}}_{m}$ is detailed in Appendix D. The results indicate that while the FB method achieves higher accuracy in estimating the latent variable ${\vec{U}}_{m}$, the GA method offers greater convenience and time-efficiency. Additionally, the effects of these two methods on model parameter estimation and regression analysis are assessed through simulation studies in Section 3. The results reveal that both methods yield similar parameter estimates; however, the FB method produces fitting results with higher accuracy, a benefit attributed to its superior estimation of the latent variable. In conclusion, the FB method is suitable when the number of data points is small, while the GA method is recommended for larger datasets.

#### 2.2.4. Generate $\Theta ∼p\left(\Theta |\vec{Y},\vec{Z},\vec{U}\right)$

In Step 3, parameters are partitioned into three blocks according to the model structure. The posterior distribution of the parameters can be expressed as follows:

$$\begin{array}{cc} \; & p\left(\Theta |\vec{Y},\vec{Z},\vec{U}\right)\\ = & p\left(a,b,c,\alpha ,\sigma_{\epsilon }^{2}|\vec{Y},\vec{Z}\right)\cdot p\left(\theta_{K}|\vec{Z},\vec{U}\right)\cdot p\left(\tau ,\mu |\vec{U}\right)\\ \propto & \left[p\left(\vec{Y}|\vec{Z},a,b,c,\alpha ,\sigma_{\epsilon }^{2}\right)p\left(a,b,c,\alpha ,\sigma_{\epsilon }^{2}\right)\right]\cdot \left[p\left(\vec{Z}|\vec{U},\theta_{K}\right)p\left(\theta_{K}\right)\right]\cdot \left[p\left(\vec{U}|\tau ,\mu \right)p\left(\tau ,\mu \right)\right]\\ = & \left[\prod_{m=1}^{M}p\left({\vec{Y}}_{m}|{\vec{Z}}_{m},a,b,c,\alpha ,\sigma_{\epsilon }^{2}\right)p\left(a,b,c,\alpha ,\sigma_{\epsilon }^{2}\right)\right]\\ \; & \;\cdot \left[\prod_{m=1}^{M}p\left({\vec{Z}}_{m}|{\vec{U}}_{m},\theta_{K}\right)p\left(\theta_{K}\right)\right]\cdot \left[\prod_{m=1}^{M}p\left({\vec{U}}_{m}|\tau ,\mu \right)p\left(\tau ,\mu \right)\right]\\ = & \left[\prod_{m=1}^{M}N\left(\left[RA⊗I_{n_{m}}\right]{\vec{Z}}_{m}+c⊗1_{n_{m}},\;\sigma_{\epsilon }^{2}I_{2n_{m}}\right)p\left(a,b,c,\alpha ,\sigma_{\epsilon }^{2}\right)\right]\\ \; & \;\cdot \left[\prod_{m=1}^{M}N\left({\vec{U}}_{m},\;I_{2}⊗K_{m}\right)p\left(\theta_{K}\right)\right]\cdot \left[\prod_{m=1}^{M}\mathrm{vMF}\left(\tau ,\mu \right)p\left(\tau ,\mu \right)\right]. \end{array}$$

The prior distributions for the unknown parameters should be specified for Bayesian inference. The rotation of the ellipse, α, is constrained to cover a 90-degree range to ensure the uniqueness of the solution, preventing the switching of major and minor axes. The mean direction μ is assigned a vMF distribution [40]. If a uniform prior on the unit circle is desired, the hyperparameter τ in the prior distribution of μ can be set to zero. For the remaining parameters, truncated Gaussian priors to items constrained to be positive, while general Gaussian priors are used for unconstrained parameters. The hyperparameters for these priors are either predefined or based on initialization, with the mean of the Gaussian prior typically set to the initial value of each parameter. The initialization of these parameters will be discussed later.

The general random-walk Metropolis–Hastings algorithm [69,70] is utilized in Step 3 to generate random parameters. Symmetric proposal distributions (Gaussian or truncated Gaussian distribution) are employed for each parameter based on previously sampled values, ensuring that $p\left(\Theta^{\left(s+1\right)}|\Theta^{\left(s\right)}\right)=p\left(\Theta^{\left(s\right)}|\Theta^{\left(s+1\right)}\right)$, then the acceptance probabilities correspond to the ratio of the posterior densities. To illustrate the Metropolis–Hastings algorithm, we consider the parameters in one block, $\left(a,b,c,\alpha ,\sigma_{\epsilon }^{2}\right)$. After generating candidate values, the acceptance ratio is calculated as follows:

$$\begin{array}{cc} \mathrm{ar} & =\frac{p\left(a^{*},b^{*},c^{*},\alpha^{*},\sigma_{\epsilon }^{*},\nu^{2(s)},\omega^{2(s)},\tau^{\left(s\right)},\mu^{\left(s\right)}|\vec{Y},\vec{Z},\vec{U}\right)}{p\left(a^{\left(s\right)},b^{\left(s\right)},c^{\left(s\right)},\alpha^{\left(s\right)},\sigma_{\epsilon }^{2(s)},\nu^{2(s)},\omega^{2(s)},\tau^{\left(s\right)},\mu^{\left(s\right)}|\vec{Y},\vec{Z},\vec{U}\right)}\\ \; & =\frac{\prod_{m=1}^{M}p\left({\vec{Y}}_{m}|{\vec{Z}}_{m},a^{*},b^{*},c^{*},\alpha^{*},\sigma_{\epsilon }^{*}\right)p\left(a^{*},b^{*},c^{\left(s\right)},\alpha^{\left(s\right)},\sigma_{\epsilon }^{2(s)}\right)}{\prod_{m=1}^{M}p\left({\vec{Y}}_{m}|{\vec{Z}}_{m},a^{\left(s\right)},b^{\left(s\right)},c^{\left(s\right)},\alpha^{\left(s\right)},\sigma_{\epsilon }^{2(s)}\right)p\left(a^{\left(s\right)},b^{\left(s\right)},c^{\left(s\right)},\alpha^{\left(s\right)},\sigma_{\epsilon }^{2(s)}\right)}. \end{array}$$

A uniform random value is then generated; if this value is less than or equal to ar, the candidate is accepted by setting $\left(a^{\left(s+1\right)},b^{\left(s+1\right)},c^{\left(s+1\right)},\alpha^{\left(s+1\right)},\sigma_{\epsilon }^{2(s+1)}\right)=\left(a^{*},b^{*},c^{*},\alpha^{*},\sigma_{\epsilon }^{*}\right)$. Otherwise, it is rejected, and $(a^{\left(s+1\right)},b^{\left(s+1\right)},c^{\left(s+1\right)},\alpha^{\left(s+1\right)}$$,\sigma_{\epsilon }^{2(s+1)})=\left(a^{\left(s\right)},b^{\left(s\right)},c^{\left(s\right)},\alpha^{\left(s\right)},\sigma_{\epsilon }^{2(s)}\right)$.

After $s_{0}$ initial steps (the “warming up” period), the random variables ${\left\{\Theta^{\left(s\right)},{\vec{Z}}^{\left(s\right)},{\vec{U}}^{\left(s\right)}\right\}}_{s=s_{0}+1}^{s_{0}+S}$ can be regarded as approximately distributed according to the joint distribution $p\left(\Theta ,\vec{Z},\vec{U}|\vec{Y}\right)$ as $s_{0}+S\to +\infty$, under mild regularity conditions [71]. The unknown variables $\vec{Z}$, $\vec{U}$, and parameters Θ are estimated based on the posterior mean. Moreover, the hyperparameter $\theta_{K}$ can also be estimated within an empirical Bayesian framework, such as by maximizing the Laplace approximated marginal likelihood. The estimator is consistent when *M* is sufficiently large and $n=o(M^{2})$ under certain regularity conditions, as discussed in [43] and proved in Appendix E.

#### 2.2.5. Initialization

The initialization process for the parameters and latent variables is crucial for the performance of the sampling procedure. Initial values are denoted with the subscript “init”. The ellipse-related parameters are initialized using the least-squares ellipse fitting algorithm described in [13], with code adapted by Michael Bedward in r-help. This algorithm is applied to the points in each curve, and the average is taken as the initial value. For the latent variables, we set

$$\begin{array}{cc} {\vec{Z}}_{\mathrm{init},m} & =\left[A_{\mathrm{init}}^{-1}R_{\mathrm{init}}^{T}⊗I_{n_{m}}\right]\left[{\vec{Y}}_{m}-c_{\mathrm{init}}⊗1_{n_{m}}\right],\;m=1,\;\cdots ,\;M,\\ u_{\mathrm{init},mi} & =z_{\mathrm{init},mi}/{∥z_{\mathrm{init},mi}∥}_{2},\;i=1,\;\cdots ,\;n_{m}, \end{array}$$

where

$$R_{\mathrm{init}}=\left(\begin{array}{cc} \cos\alpha_{\mathrm{init}} & -\sin\alpha_{\mathrm{init}}\\ \sin\alpha_{\mathrm{init}} & \cos\alpha_{\mathrm{init}} \end{array}\right),\;A_{\mathrm{init}}=\left(\begin{array}{cc} a_{\mathrm{init}} & 0\\ 0 & b_{\mathrm{init}} \end{array}\right),$$

which refer to Equation (4). Here, ${\vec{Z}}_{\mathrm{init},m}$ is the mapped data to be around the unit circle, and $u_{\mathrm{init},mi}$ is the scaled $z_{\mathrm{init},mi}$ that is initialized to lie on the unit circle. The initial value of the noise variance $\sigma_{\epsilon \mathrm{init}}^{2}$ is set to the sample mean of the variance of $\left({\vec{Y}}_{m}-\left[R_{\mathrm{init}}A_{\mathrm{init}}⊗I_{n_{m}}\right]{\vec{U}}_{\mathrm{init},m}-c_{\mathrm{init}}⊗1_{n_{m}}\right)$. For the hyperparameters, $\nu_{\mathrm{init}}$ is initialized to the sample mean of variance of ${\vec{Z}}_{\mathrm{init},m}$, and $\omega_{\mathrm{init}}$ is set to the square of the smallest timespan length. The value of $\mu_{\mathrm{init}}$ is determined as the scaled sample mean of ${\tilde{\mu }}_{\mathrm{init},m}$, where ${\tilde{\mu }}_{\mathrm{init},m}=\sum_{i=1}^{n_{m}}u_{\mathrm{init},mi}/\left|\sum_{i=1}^{n_{m}}u_{\mathrm{init},mi}\right|$ is the scaled average for *m*-th batch. The $\tau_{\mathrm{init}}$ is initialized to be the sample mean of the proposed estimator from [72]. The equations are defined as follows:

$$\begin{array}{cc} \mu_{\mathrm{init}} & =\sum_{m=1}^{M}{\tilde{\mu }}_{\mathrm{init},m}/\left|\sum_{m=1}^{M}{\tilde{\mu }}_{\mathrm{init},m}\right|,\\ \tau_{\mathrm{init}} & =\frac{1}{M}\sum_{m=1}^{M}0.6724^{2}/\mathrm{median}\left(2\cdot \left(1-{\tilde{\mu }}_{\mathrm{init},m}^{T}u_{\mathrm{init},mi}\right)\right). \end{array}$$

## 3. Simulation Studies

Simulation studies were conducted to evaluate the proposed model’s performance. Observational data were generated, consisting of *M* batches, each containing *n* data points. The time range T was set to [0,1] with evenly spaced time points. To transform the unit circle into an ellipse, the parameters related to the ellipse were set as a=20, b=10, $c={\left(1,1\right)}^{T}$, and α=π/4. The first simulation aims to demonstrate the accuracy of parameter estimation and the goodness of fit for the observational data, aligning the data generation settings with the proposed model structure. The second simulation focused on evaluating the accuracy of an additional model in parameter estimation.

Parameter estimation was performed using the MCMC method with 10,000 iterations ($\vec{S}+s_{0}$ = 10,000). The first half of the iterations was designated as the burn-in period, i.e., $s_{0}=5000$. To reduce the correlation between samples, recordings were made at intervals of 20 iterations. Posterior means were calculated from the post-burn-in samples. The convergence of the MCMC chains is examined using the potential scale reduction factor (PSRF) [73,74], detailed in Appendix G. The PSRF has been computed for each parameter, and the cutoff 1.1, i.e., PSRF ≤1.1, is used to determine convergence [33,75]. Convergence is deemed satisfactory.

### 3.1. Simulation 1

In the first simulation, two scenarios were considered: Scenario 1, with τ=0.01, representing an almost complete elliptical shape, and Scenario 2, with τ=1, depicting a partial region of the ellipse. The observational data were generated as y(t)=RAz(t)+c+ϵ(t), where $z\left(t\right)=u\left(t\right)+1_{2}⊗G\left(t\right)$ and $\epsilon \left(t\right)∼N\left(0,0.1I_{2}\right).$ The two scenarios are defined as

Scenario 1: $u\left(t\right)∼\mathrm{vMF}({\left(1,0\right)}^{T},0.01),$
Scenario 2: $u\left(t\right)∼\mathrm{vMF}({\left(1,0\right)}^{T},1).\;\;$

Here, G(t) is a GP with zero mean and a squared exponential covariance structure (see Equation (2)), with parameters ν=0.01 and $\omega =10^{-4}$. The small value of ω was chosen based on the time range and the shortest timespan. In our real data, the timespan is typically 0.01 seconds. By selecting ω as $10^{-4}$, we retain the necessary within-curve dependency while ensuring a non-zero multivariate Gaussian probability for ${\vec{Z}}_{m}$.

Different combinations of *M* (the number of batches, set to 10 or 30) and *n* (the number of data points per batch, set to 10 or 30) were tested. The accuracy and precision of parameter estimation were assessed using root mean square error (RMSE) and standard deviation (SD) calculated from the parameter estimates across 100 replications and the true values. These results are presented in Table 1. Different methods for sampling $\vec{U}$, including GA and FB methods, were compared. The precision of parameter estimation improved, as indicated by smaller RMSE and SD, with increasing *n* and *M*. In Scenario 1, the estimation of μ exhibited increased fluctuation with larger *n* or *M* values, which can be deemed acceptable given the almost complete elliptical shape and the mean position for u(t) can actually be in any direction. In Scenario 2, where τ=1 and the data points represented only a portion of the ellipse, the estimation accuracy of μ improved with increasing *n* and *M*. GA method exhibits similar parameter estimation performance compared to FB. Moreover, a detailed investigation of the effect of τ on parameter estimation was conducted and is presented in Appendix F. Notably, when τ exceeds 2, a significant bias in the estimation of elliptical parameters is observed, attributed to the substantial deviation of the initial estimates from their true values, which makes convergence challenging in subsequent iterations.

The difference between the fitted values and the observations was evaluated using the RMSE and mean absolute error (MAE) as goodness-of-fit measures. Table 2 shows the sample mean of RMSE and MAE based on 100 replications. The fitting performances improved as *n* and *M* increased, as expected. FB approach for sampling $\vec{U}$ outperformed GA with smaller average RMSE and MAE as *n* and *M* increased.

### 3.2. Simulation 2

In the additional model, subject-specific information was integrated into the parameters defining the ellipse, allowing variability among subjects to be reflected in the parameter estimates. A relationship was established such that $\alpha \left(x_{m}\right)=\alpha_{0}+\alpha_{1}x_{m}$, with $x_{m}$ representing an indicator variable. Specifically, $x_{m}=1$ for subject *m* in group 1, and $x_{m}=0$ for subject in group 2. In this simulation, the parameters were set to $\alpha_{0}=\pi /8$ and $\alpha_{1}=\pi /4$. The study generated M/2 curves for each group, with each curve consisting of *n* data points. All other parameters remained consistent with those used in Simulation 1. Given the previously demonstrated comparability of results between the GA and FB methods, this simulation focuses on presenting the results from the GA method. Table 3 reports the RMSE and SD for each parameter based on 100 replications. The results demonstrate enhanced accuracy in parameter estimation with increasing *n* and *M*. In comparison to Simulation 1, the parameters related to the ellipse shape were estimated with greater accuracy, as expected. However, a trade-off was observed, manifesting as a slight increase in the error associated with estimating $\sigma_{\epsilon }^{2}$, which can be attributed to model simplification.

## 4. Applications in Gait Analysis

### 4.1. Center of Mass Excursion

The phase plot technique is an important tool for examining oval-shaped data, as discussed in Section 1. Given a signal h(t) over time points $t_{i}$, i=1,2,⋯,n, a phase plot of h(t) is created by plotting $y_{2t}=h\left(t\right)$ against $y_{1t}=h\left(t-1\right)$, as illustrated in the right panel of Figure 3. When periodicity is evident in the signal, a smoothed curve can be generated to produce an orbital plot. This method has gained considerable traction in the gait analysis literature, primarily focusing on constructing an ellipse to encapsulate the spread of the cloud shape [1,2,3].

In this study, the phase plot of the CoM excursion data from dementia patients during continuous walking was employed. The acceleration signals were recorded using a low-cost wearable sensor AX6 (Axivity Ltd., Newcastle upon Tyne, UK; 2.3×3.3×0.8 cm, 11 g), which features a configurable tri-axial accelerometer (±2–16 g) and tri-axial gyroscope (125–2000° per second, dps) sensor BMI160 (Bosch Sensortec, Reutlingen, Germany) with variable sampling capabilities (e.g., 50 or 100 Hz), set via the proprietary software OmGui V1.0.0.43. For this study, the AX6 was configured to ±8 g, 250 dps, and 100 Hz [76]. Participants wore the device on the fifth lumbar vertebra (approximating the CoM) [77,78] for several days in free-living environments [79]. The raw signals were segmented during preprocessing into four straight-line passes using timestamps, then transformed and filtered [80]. Walking bouts need to be detected first, followed by the detection of gait cycles based on the peak of the signal [81].

**Figure 4:**
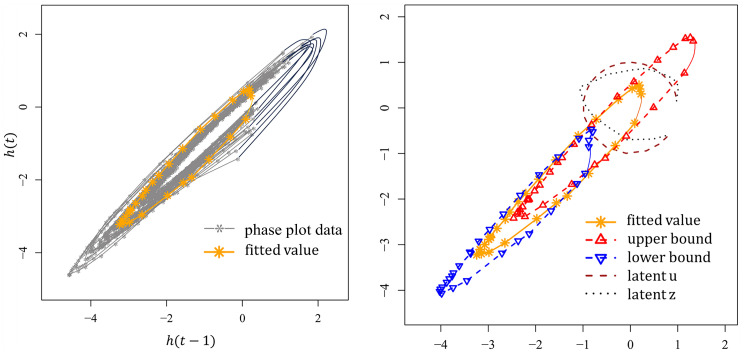
Visualization of the proposed model’s performance in fitting phase plot data. (**Left panel**) The curve fitted by the proposed model to the observed phase plot data. (**Right panel**) Fitted values with corresponding 95% confidence intervals (upper and lower bounds), along with the estimated latent variables ***z*** and ***u***.

In this analysis, the focus was restricted to gait cycles with a duration range from 0.5 to 0.7 s within a 69-s walking bout. To expedite the estimation process, 31 data points were randomly sampled for each cycle, resulting in irregularly spaced CoM data. The aim was for the proposed model to reasonably depict the dynamics of the structure. The estimation results, displayed in Figure 4, demonstrate that the curve fitted by the model effectively captures the shape of the phase plot. The estimated parameters are as follows: a=2.4007, b=0.4114, $c={\left(-1.3371,-1.4503\right)}^{T}$, α=0.8383 (in radians), τ=1.2691, $\mu ={\left(-0.6054,0.7959\right)}^{T}$, ν=0.0890, $\omega =8.6281\times 10^{-5}$, and $\sigma_{\epsilon }^{2}=0.0068$. The proposed model allows for the estimation of confidence intervals, along with the latent variables ***z*** and ***u***, where ***z*** represents the trajectory of systematic error in the time-dependent process, varying around ***u***, which is located on a unit circle.

### 4.2. Freezing of Gait Detection

**Figure 5:**
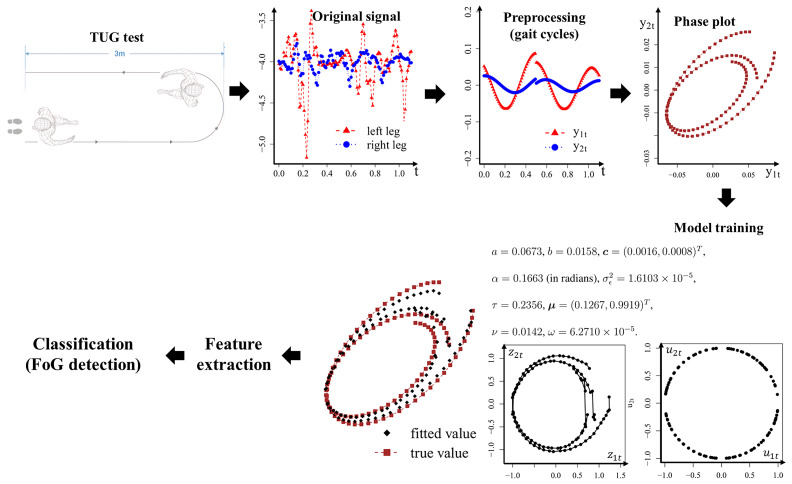
Flow chart representation of the analytical procedure for FoG detection.

Detecting FoG presents challenges in gait analysis. FoG is characterized by a sudden and temporary inability to initiate or continue walking. Recent technological advancements, including wearable sensors and deep brain stimulation, offer avenues for managing and mitigating this complex symptom [82,83]. In a controlled laboratory setting, wearable devices were utilized to collect data regarding freezing gait from 15 patients diagnosed with Parkinson’s disease during the timed up-and-go (TUG) test [84]. The TUG test entails patients walking in a straight line from a starting position, turning around, and returning to the starting point. In this study, patients with fewer than three freezing cycles were filtered, resulting in a final sample of 8 patients. An overview of our analytical procedure for FoG detection is presented in Figure 5.

In our analysis, $y_{1t}$ is the continuous wavelet-transformed vertical acceleration captured by a sensor placed on the left leg, while $y_{2t}$ refers to the preprocessed signal obtained from another sensor placed on the right leg. The data were segmented into cycles based on the first derivative and identified stationary points of the curve, followed by centering each curve. The phase plot of $y_{2t}$ versus $y_{1t}$ for each cycle exhibits an elliptical shape. Although the fitted curves exhibited deviations attributed to significant differences between the curves of the two legs, the fitted results nonetheless provided a reasonable approximation of the overall shape. Previous analyses revealed that the parameters estimated during the freezing period were significantly smaller compared to those in the non-freezing phase, reflecting the short, shuffling steps characteristic of FoG [85]. The ellipse parameters, the concentration of the latent variable ***u***, and the noise variance displaced noticeable decreases during freezing episodes.

Following model training, elliptical features were extracted for subsequent FoG detection. The parameters *a* and *b* estimated from the model possess interpretations related to the ellipse, corresponding to the semi-major and semi-minor axes, respectively. Using these parameters, a series of ellipse-related features were computed, including eccentricity (e), conjugate diameter (d), and semi-latus rectum (r). The formulas for these features are presented in Table 4. Furthermore, the covariance matrices of the elliptical features were calculated for both freezing and non-freezing cycles, with the upper triangular elements of the matrix representing the non-freezing cycles and the lower elements (in parentheses) representing the freezing cycles.

For the final classification modeling, the extracted elliptical features and model parameters were utilized as input variables, employing the leave-one-person-out cross-validation method to evaluate the FoG detection performance. The results, summarized in Table 5, indicate that the precision of both support vector machine (SVM) and random forest (RF) classification methods, utilizing the extracted elliptical features and our model parameters, approached approximately 0.8, surpassing the recognition accuracy reported in a prominent study by [86] (Gyenno’s research). It is noteworthy that we utilized only 16 features, substantially lower than the feature set of 624 employed in Gyenno’s research. Furthermore, our methodology relied solely on acceleration signals from the left and right legs, necessitating only two sensors, whereas most contemporary studies typically require seven sensors: one at the waist and six at the legs [87]. Thus, the proposed model not only excels in accuracy but also conserves resources by requiring fewer sensors and extracting a lower-dimensional feature set.

## 5. Discussion

The proposed model offers advantages in capturing curvilinear dependencies for high-dimensional data. Its primary strengths lie in the utilization of the vMF distribution and the incorporation of GP prior. The vMF distribution provides a straightforward parametric representation of the underlying elliptical shape, and the GP allows for modeling point dependency and systematic error through a specific covariance structure.

Beyond its methodology, the proposed model possesses exploratory value in gait analysis. The motivating examples yield detailed insights into gait coordination and stability by examining the phase plot of the CoM excursion and FoG data. Such in-depth analyses enable the identification of subtle variations in gait patterns that may not be apparent using traditional techniques. Furthermore, the variation in the shape of the phase plot across different walking bout lengths offers additional insights into gait assessment and potential intervention areas. The model’s parameters, which capture the gait pattern over time, can be related to pathological characteristics, assisting in the screening and diagnosis of gait-related disorders.

While demonstrating the model’s utility in gait analysis, several avenues for further development remain. Future investigations could consider implementing models with varying coefficients. For instance, in longitudinal studies where $\alpha_{m}\left(s\right)=\beta_{\alpha }\left(s\right)x_{m}$, the parameter $\beta_{\alpha }\left(s\right)$ could explore gait changes correlated with advancing age potentially elucidating the progression of neurodegenerative disorders. Additionally, in cases where data exhibit more complex shapes than simple ellipses, alternative high-dimensional directional distributions might be more suitable. The exploration of non-Gaussian distributions or correlated measurement errors across data points may also necessitate refinement in model specification. Computational efficiency remains an area for ongoing research. While sampling and MCMC methods yield accurate estimators, the need for more efficient approaches warrants attention. This study explores the GA method for sampling from the FB distribution; however, further development of statistical theories within this context is necessary.

## Figures and Tables

**Table 1:** Precision of parameter estimation in Simulation 1: RMSE and SD (in parentheses) of parameter estimates across GA and FB sampling methods for $\vec{U}$, based on 100 replications.

True Values	M=10, n=10		M=10, n=30		M=30, n=30	
Scenario 1	GA	FB	GA	FB	GA	FB
a=20	0.5361(0.5135)	0.5401(0.5177)	0.2666(0.2280)	0.3081(0.2919)	0.2267(0.1321)	0.2252(0.1296)
b=10	0.3068(0.2910)	0.3081(0.2919)	0.1421(0.1208)	0.1369(0.1167)	0.0900(0.0574)	0.0893(0.0584)
$c_{1}=1$	0.3706(0.3724)	0.3671(0.3688)	0.1533(0.1540)	0.1487(0.1494)	0.0840(0.0828)	0.0834(0.0823)
$c_{2}=1$	0.4089(0.4109)	0.4049(0.4069)	0.1480(0.1488)	0.1509(0.1517)	0.0825(0.0827)	0.0795(0.0798)
α=π/4	0.0263(0.0263)	0.0255(0.0256)	0.0128(0.0125)	0.0138(0.0136)	0.0074(0.0074)	0.0078(0.0077)
τ=0.01	0.6334(0.1991)	0.6336(0.1999)	0.3373(0.0349)	0.3372(0.0349)	0.3391(0.0222)	0.3390(0.0222)
$\mu_{1}=1$	1.2198(0.7213)	1.2278(0.7240)	1.0861(0.5914)	1.0852(0.5901)	1.1106(0.6736)	1.1058(0.6696)
$\mu_{2}=0$	0.6962(0.6179)	0.6936(0.5727)	0.8038(0.7080)	0.8048(0.7004)	0.7333(0.7150)	0.7364(0.7214)
$\sigma_{\epsilon }^{2}=0.1$	0.0043(0.0021)	0.0039(0.0020)	0.0045(0.0024)	0.0047(0.0026)	0.0043(0.0015)	0.0036(0.0013)
Scenario 2	GA	FB	GA	FB	GA	FB
a=20	1.5685(1.1493)	1.5660(1.1485)	0.3765(0.3513)	0.3774(0.3530)	0.2136(0.1918)	0.2126(0.1919)
b=10	0.6597(0.4398)	0.6572(0.4424)	0.1496(0.1465)	0.1488(0.1475)	0.0625(0.0628)	0.0622(0.0625)
$c_{1}=1$	1.4506(0.8660)	1.4490(0.8669)	0.4073(0.2458)	0.4092(0.2488)	0.3384(0.1442)	0.3364(0.1413)
$c_{2}=1$	1.4835(0.8441)	1.4937(0.8501)	0.3806(0.2274)	0.3862(0.2324)	0.3382(0.1421)	0.3374(0.1387)
α=π/4	0.0549(0.0546)	0.0510(0.0507)	0.0167(0.0167)	0.0160(0.0160)	0.0100(0.0101)	0.0098(0.0099)
τ=1	0.3458(0.3410)	0.3429(0.3382)	0.2128(0.1115)	0.2131(0.1120)	0.1624(0.0804)	0.1628(0.0803)
$\mu_{1}=1$	0.0194(0.0162)	0.0193(0.0160)	0.0021(0.0019)	0.0021(0.0020)	0.0030(0.0026)	0.0029(0.0025)
$\mu_{2}=0$	0.1454(0.0947)	0.1465(0.0946)	0.0393(0.0352)	0.0392(0.0344)	0.0569(0.0571)	0.0562(0.0565)
$\sigma_{\epsilon }^{2}=0.1$	0.0035(0.0022)	0.0034(0.0022)	0.0045(0.0022)	0.0041(0.0024)	0.0040(0.0015)	0.0035(0.0015)

**Table 2:** Goodness-of-fit measures for Simulation 1: Mean values of RMSE and MAE for the observations, calculated from 100 replications.

	M=10, n=10		M=10, n=30		M=30, n=30	
Scenario 1	GA	FB	GA	FB	GA	FB
RMSE	0.2098	0.2138	0.1517	0.1404	0.1503	0.1389
MAE	0.1616	0.1650	0.1134	0.1047	0.1122	0.1034
Scenario 2	GA	FB	GA	FB	GA	FB
RMSE	0.1841	0.1860	0.1459	0.1350	0.1449	0.1338
MAE	0.1387	0.1400	0.1082	0.0998	0.1073	0.0989

**Table 3:** Precision of parameter estimation in Simulation 2: RMSE and SD (in parentheses) of parameter estimates using GA method for sampling $\vec{U}$, based on 100 replications.

True Values	M=10,n=10	M=10,n=30	M=30,n=30	M=30,n=60
Scenario 1	GA	GA	GA	GA
a=20	0.1913(0.1858)	0.0848(0.0764)	0.0751(0.0634)	0.0798(0.0754)
b=10	0.1163(0.1168)	0.0705(0.0708)	0.0721(0.0724)	0.0649(0.0647)
$c_{1}=1$	0.1447(0.1453)	0.0792(0.0786)	0.0732(0.0735)	0.0717(0.0720)
$c_{2}=1$	0.1373(0.1379)	0.0799(0.0801)	0.0865(0.0867)	0.0681(0.0680)
$\alpha_{0}=\pi /8$	0.0938(0.0094)	0.0943(0.0030)	0.0942(0.0024)	0.0941(0.0017)
$\alpha_{1}=\pi /4$	0.1919(0.0147)	0.1896(0.0050)	0.1890(0.0033)	0.1898(0.0024)
τ=0.01	0.5965(0.2058)	0.3212(0.0315)	0.3353(0.0205)	0.2887(0.0121)
$\mu_{1}=1$	0.4585(0.1202)	0.6686(0.0344)	0.6510(0.0218)	0.6926(0.0150)
$\mu_{2}=0$	0.8216(0.1363)	0.9426(0.0127)	0.9367(0.0082)	0.9514(0.0049)
$\sigma_{\epsilon }^{2}=0.1$	0.0415(0.0085)	0.0431(0.0084)	0.0425(0.0078)	0.0411(0.0075)
Scenario 2	GA	GA	GA	GA
a=20	0.8316(0.6718)	0.1164(0.0988)	0.1031(0.0844)	0.0871(0.0780)
b=10	0.2624(0.2141)	0.0690(0.0693)	0.0778(0.0782)	0.0625(0.0625)
$c_{1}=1$	0.6216(0.5184)	0.1139(0.1025)	0.1063(0.0805)	0.0712(0.0675)
$c_{2}=1$	0.7070(0.5772)	0.1012(0.0940)	0.0913(0.0763)	0.0841(0.0741)
$\alpha_{0}=\pi /8$	0.0976(0.0416)	0.0943(0.0042)	0.0942(0.0026)	0.0947(0.0021)
$\alpha_{1}=\pi /4$	0.2110(0.0564)	0.1905(0.0057)	0.1892(0.0042)	0.1893(0.0029)
τ=1	0.5719(0.4706)	0.2081(0.1110)	0.1570(0.0676)	0.1944(0.0377)
$\mu_{1}=1$	0.0816(0.0641)	0.2758(0.1141)	0.2236(0.0782)	0.3562(0.0364)
$\mu_{2}=0$	0.3092(0.2552)	0.6531(0.2071)	0.6077(0.1297)	0.7628(0.0312)
$\sigma_{\epsilon }^{2}=0.1$	0.0415(0.0083)	0.0420(0.0080)	0.0429(0.0087)	0.0416(0.0083)

**Table 4:** Elliptical features derived from the proposed model and their corresponding covariance matrices for freezing (upper triangular) and non-freezing (lower triangular in parentheses) gait cycles.

Features	Formula	*a*	*b*	*e*	*d*	*r*
*a*		15.0436 (3.6854)	2.5321	0.1274	0.2144	−0.0531
*b*		(0.9443)	5.1933 (0.4224)	−0.2544	−0.4041	8.1723
*e*	$|a^{2}-b^{2}|/a$	(0.0533)	(−0.0164)	0.0214 (0.0145)	0.0316	-0.6323
*d*	$e^{2}-1$	(0.0823)	(−0.0234)	(0.0237)	0.0525 (0.0314)	−0.0824
*r*	$b^{2}/\left|a^{2}-b^{2}\right|$	(0.2524)	(0.2436)	(−0.0324)	(−0.0633)	20.2123 (0.2346)

**Table 5:** Comparative analysis of FoG detection performance using different classification models and the leave-one-person-out cross-validation method.

Model	Accuracy	Recall	Specificity	Precision	F-Score
SVM	0.8663	0.7774	0.8524	0.7986	0.7674
RF	0.8613	0.7855	0.8444	0.8023	0.7661
Gyenno	0.8797	0.7834	0.9165	0.7753	0.7793

**Figure A1:**
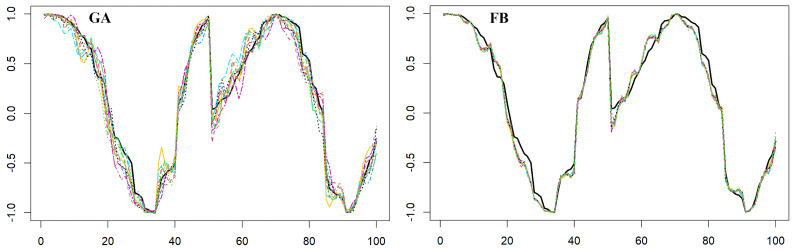
Ten random samples ${\vec{U}}_{m}$ (with $n_{m}=50$) were generated under the true parameters $\Theta_{0}$ and the true latent variable ${\vec{Z}}_{m0}$. Dashed lines in different colors represent individual random samples, while the solid black line indicates the true ${\vec{U}}_{m0}$. (**Left panel**) Samples generated by the GA method. (**Right panel**) Samples generated by the FB method.

**Figure A2:**
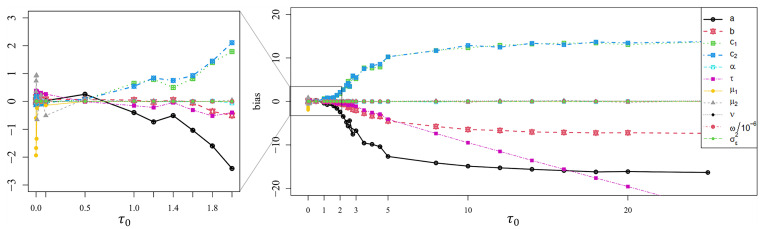
Bias for each estimated parameter with respect to different $\tau_{0}$ values.

**Figure A3:**
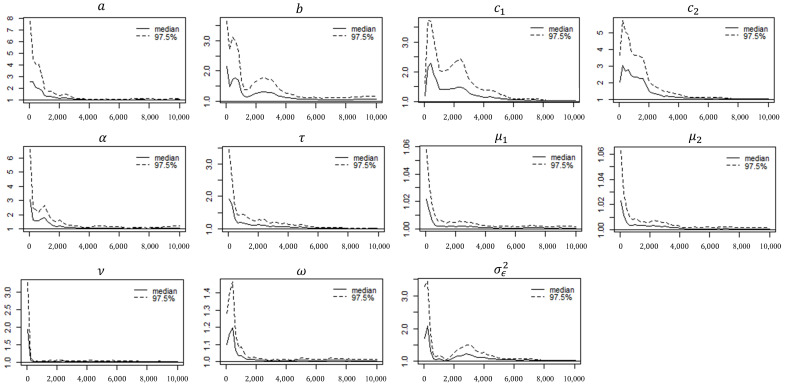
Median PSRF and its 97.5% quantile versus iterations under five chains for each parameter.

## Data Availability

The manuscript used two real-world datasets: (1) Center of mass excursion and (2) Freezing of gait detection. The first data set is publicly available and can be downloaded via https://www.jianguoyun.com/p/DZB2eM4Q9NP8DBi7htoFIAA (accessed on 12 October 2024). The second data set is available upon request, which is used in the second example illustrating the performance of the proposed statistical methods. This is part of the data in our ongoing joint project.

## Appendix A. VMF and FB Distributions

The vMF distribution is a probability distribution defined on the (p−1)-sphere in ${ℜ}^{p}$. Its density function is expressed as follows:

$$p_{p}\left(x;\mu ,\tau \right)=C_{k}\left(\tau \right)\exp\left(\tau \mu^{T}x\right),\;∥x∥=1,$$

where $\mu \in {ℜ}^{p}$ indicates the mean position (direction), and τ≥0 is the scale parameter that controls the concentration of the distribution around the mean direction μ [44]. The normalization constant $C_{p}\left(\tau \right)$ is given by

$$C_{p}\left(\tau \right)=\frac{\tau^{p/2-1}}{{\left(2\pi \right)}^{p/2}I_{p/2-1}\left(\tau \right)},$$

where $I_{v}\left(\tau \right)$ is the modified Bessel function of the first kind of order v, defined as

$$I_{v}\left(\tau \right)=\frac{1}{2\pi }\int_{0}^{2\pi }e^{\tau \cos t}\cos\left(vt\right)dt.$$

In the two-dimensional case, where p=2 and $u_{t}\in {ℜ}^{2\times 1}$ represents a point on the unit circle, the density function can be written as

$$\begin{array}{c} p\left(u_{t};\mu ,\tau \right)=C_{2}\left(\tau \right)\exp\left(\tau \mu^{T}u_{t}\right)=\frac{1}{2\pi I_{0}\left(\tau \right)}\exp\left(\tau \mu^{T}u_{t}\right). \end{array}$$

The expected value and covariance for this distribution are given by

$$\begin{array}{c} E\left(u_{t}\right)=\frac{I_{1}\left(\tau \right)}{I_{0}\left(\tau \right)}\mu ,\;Cov\left(\mu_{t}\right)=\frac{1}{\tau }\frac{I_{1}\left(\tau \right)}{I_{0}\left(\tau \right)}I_{2}+\left\{1-\frac{2}{\tau }\frac{I_{1}\left(\tau \right)}{I_{0}\left(\tau \right)}-{\left(\frac{I_{1}\left(\tau \right)}{I_{0}\left(\tau \right)}\right)}^{2}\right\}\mu \mu^{T}. \end{array}$$

A random vector x (with ∥x∥=1) that follows the FB distribution has the density given by

(A1) $$\mathrm{FB}\left(x;\kappa \vartheta ,B\right)=\frac{1}{\zeta (\kappa \vartheta ,B)}\exp\left(\kappa \vartheta^{T}x-x^{T}Bx\right),$$

where x, ϑ∈(p−1)-sphere, κ≥0, $B\in {ℜ}^{p\times p}$ is a symmetric matrix, and ζ(κϑ,B) is a normalizing constant [61].

## Appendix B. Proof of the Conditional Distribution in Step 1

The distribution of ${\vec{Z}}_{m}$ given ${\vec{Y}}_{m}$, ${\vec{U}}_{m}$, and all other parameters is proportional to the product of two Gaussian distributions, as follows:

$$\begin{array}{cc} \; & p\left({\vec{Z}}_{m}|{\vec{Y}}_{m},{\vec{U}}_{m},\Theta \right)\\ \propto & p\left({\vec{Y}}_{m}|{\vec{Z}}_{m},{\vec{U}}_{m},\Theta \right)\cdot p\left({\vec{Z}}_{m}|{\vec{U}}_{m},\Theta \right)\\ = & p\left({\vec{Y}}_{m}|{\vec{Z}}_{m},\Theta \right)\cdot p\left({\vec{Z}}_{m}|{\vec{U}}_{m},\Theta \right)\\ \propto & \exp\{-\frac{1}{2\sigma_{\epsilon }^{2}}{\left({\vec{Y}}_{m}-\left[RA⊗I_{n_{m}}\right]{\vec{Z}}_{m}-c⊗1_{n_{m}}\right)}^{T}\left({\vec{Y}}_{m}-\left[RA⊗I_{n_{m}}\right]{\vec{Z}}_{m}-c⊗1_{n_{m}}\right)\\ \; & \;-\frac{1}{2}{\left({\vec{Z}}_{m}-{\vec{U}}_{m}\right)}^{T}\left[I_{2}⊗K_{m}^{-1}\right]\left({\vec{Z}}_{m}-{\vec{U}}_{m}\right)\}\\ \; & =\exp\{-\frac{1}{2\sigma_{\epsilon }^{2}}{\left({\vec{Z}}_{m}-\left[A^{-1}R^{T}⊗I_{n_{m}}\right]\left[{\vec{Y}}_{m}-c⊗1_{n_{m}}\right]\right)}^{T}\left[AR^{T}⊗I_{n_{m}}\right]\left[RA⊗I_{n_{m}}\right]\\ \; & \;\left({\vec{Z}}_{m}-\left[A^{-1}R^{T}⊗I_{n_{m}}\right]\left[{\vec{Y}}_{m}-c⊗1_{n_{m}}\right]\right)-\frac{1}{2}{\left({\vec{Z}}_{m}-{\vec{U}}_{m}\right)}^{T}\left[I_{2}⊗K_{m}^{-1}\right]\left({\vec{Z}}_{m}-{\vec{U}}_{m}\right)\}.\\ \; & =\exp\{-\frac{1}{2}{\left({\vec{Z}}_{m}-\left[A^{-1}R^{T}⊗I_{n_{m}}\right]\left[{\vec{Y}}_{m}-c⊗1_{n_{m}}\right]\right)}^{T}\left[\frac{1}{\sigma_{\epsilon }^{2}}A^{2}⊗I_{n_{m}}\right]\\ \; & \;\left({\vec{Z}}_{m}-\left[A^{-1}R^{T}⊗I_{n_{m}}\right]\left[{\vec{Y}}_{m}-c⊗1_{n_{m}}\right]\right)-\frac{1}{2}{\left({\vec{Z}}_{m}-{\vec{U}}_{m}\right)}^{T}\left[I_{2}⊗K_{m}^{-1}\right]\left({\vec{Z}}_{m}-{\vec{U}}_{m}\right)\}. \end{array}$$

Let $M_{1}=\left[A^{-1}R^{T}⊗I_{n_{m}}\right]\left[{\vec{Y}}_{m}-c⊗1_{n_{m}}\right]$, $\Sigma_{1}=\sigma_{\epsilon }^{2}A^{-2}⊗I_{n_{m}}$, and $\Sigma_{2}=I_{2}⊗K_{m}$. For these two symmetric matrices, we have $\Sigma_{1}^{T}=\Sigma_{1}$ and $\Sigma_{2}^{T}=\Sigma_{2}$, allowing us to derive $\left(\Sigma_{1}^{-1}+\Sigma_{2}^{-1}\right)=\Sigma_{1}^{-1}\Sigma_{2}^{-1}\left(\Sigma_{1}+\Sigma_{2}\right)$, and have

$$\begin{array}{cc} \; & p\left({\vec{Z}}_{m}|{\vec{Y}}_{m},{\vec{U}}_{m},\Theta \right)\\ \propto & \exp\left\{-\frac{1}{2}{\left({\vec{Z}}_{m}-M_{1}\right)}^{T}\Sigma_{1}^{-1}\left({\vec{Z}}_{m}-M_{1}\right)-\frac{1}{2}{\left({\vec{Z}}_{m}-{\vec{U}}_{m}\right)}^{T}\Sigma_{2}^{-1}\left({\vec{Z}}_{m}-{\vec{U}}_{m}\right)\right\}\\ \propto & \exp\{-\frac{1}{2}{\left({\vec{Z}}_{m}-\Sigma_{1}\Sigma_{2}{\left(\Sigma_{1}+\Sigma_{2}\right)}^{-1}\left[\Sigma_{1}^{-1}M_{1}+\Sigma_{2}^{-1}{\vec{U}}_{m}\right]\right)}^{T}\\ \; & \;\Sigma_{1}^{-1}\Sigma_{2}^{-1}\left(\Sigma_{1}+\Sigma_{2}\right)\left({\vec{Z}}_{m}-\Sigma_{1}\Sigma_{2}{\left(\Sigma_{1}+\Sigma_{2}\right)}^{-1}\left[\Sigma_{1}^{-1}M_{1}+\Sigma_{2}^{-1}{\vec{U}}_{m}\right]\right)\}. \end{array}$$

Thus,

$$\begin{array}{cc} \left({\vec{Z}}_{m}|{\vec{Y}}_{m},{\vec{U}}_{m},\Theta \right) & ∼N\left(\Sigma_{1}\Sigma_{2}{\left(\Sigma_{1}+\Sigma_{2}\right)}^{-1}\left[\Sigma_{1}^{-1}M_{1}+\Sigma_{2}^{-1}{\vec{U}}_{m}\right],\;\Sigma_{1}\Sigma_{2}{\left(\Sigma_{1}+\Sigma_{2}\right)}^{-1}\right)\\ \; & =N\left(\Sigma_{zm}\left(\frac{1}{\sigma_{\epsilon }^{2}}\left[AR^{T}⊗I_{n_{m}}\right]\left[{\vec{Y}}_{m}-c⊗1_{n_{m}}\right]+\left[I_{2}⊗K_{m}^{-1}\right]{\vec{U}}_{m}\right),\;\Sigma_{zm}\right). \end{array}$$

where

$$\begin{array}{c} \Sigma_{zm}=\Sigma_{1}\Sigma_{2}{\left(\Sigma_{1}+\Sigma_{2}\right)}^{-1}=\sigma_{\epsilon }^{2}\cdot \left[A^{-2}⊗I_{n_{m}}\right]\cdot \left[I_{2}⊗K_{m}\right]\cdot {\left(\sigma_{\epsilon }^{2}\left[A^{-2}⊗I_{n_{m}}\right]+I_{2}⊗K_{m}\right)}^{-1}. \end{array}$$

## Appendix C. Proof of the Conditional Distribution in Step 2

The distribution of ${\left\{u_{mi}\right\}}_{i=1}^{n_{m}}$ given ${\vec{Y}}_{m}$, ${\vec{Z}}_{m}$, and all other parameters is proportional to the product of a Gaussian distribution with a vMF distribution, as shown below:

$$\begin{array}{cc} \; & p\left(u_{mi}|{\vec{Y}}_{m},{\vec{Z}}_{m},\Theta \right)=p\left(u_{mi}|{\vec{Z}}_{m},\Theta \right)\propto p\left(z_{mi}|u_{mi},t_{m},\Theta \right)\cdot p\left(u_{mi}|\Theta \right). \end{array}$$

To describe the conditional distribution, we denote the (i,i)-th entry of the matrix $K_{m}$ as $K_{m[i,i]}$, and the *i*-th row vector of $K_{m}$ without the *i*-th item as $K_{m[i,-i]}$. Accordingly, the *i*-th column vector of $K_{m}$ without the *i*-th item is denoted as $K_{m[-i,i]}$, with $K_{m[-i,i]}={\left(K_{m[i,-i]}\right)}^{T}$, and $K_{m[-i,-i]}$ represents the submatrix of $K_{m}$ without the (i,i)-th entry. With the GP prior assumption for z(·), we have

$$\begin{array}{c} \left(z_{mi}|u_{mi},t_{m}\right)∼N\left(u_{mi},\delta I_{2}\right), \end{array}$$

where $\delta =K_{m[i,i]}-K_{m[i,-i]}{\left(K_{m[-i,-i]}\right)}^{-1}K_{m[-i,i]}$.

Then, we can express

$$\begin{array}{cc} p\left(u_{mi}|{\vec{Y}}_{m},{\vec{Z}}_{m},\Theta \right) & \propto p\left(z_{mi}|u_{mi},t_{m},\Theta \right)\cdot p\left(u_{mi}|\Theta \right)\\ \; & \propto \exp\left\{-\frac{1}{2\delta }{\left(z_{mi}-u_{mi}\right)}^{T}\left(z_{mi}-u_{mi}\right)+\tau \mu^{T}u_{mi}\right\}\\ \; & \propto \exp\left\{\left(\tau \mu^{T}+\frac{1}{\delta }z_{mi}^{T}\right)u_{mi}-\frac{1}{2\delta }u_{mi}^{T}u_{mi}\right\}. \end{array}$$

Referring to the density equation for the FB distribution in Equation (A1), we set $\kappa \vartheta =\tau \mu +\frac{1}{\delta }z_{mi}$ and $B=\frac{1}{2\delta }I_{2}$, which leads us to $\left(u_{mi}|{\vec{Z}}_{m},\Theta \right)∼\mathrm{FB}\left(u_{mi};\tau \mu +\frac{1}{\delta }z_{mi},\frac{1}{2\delta }I_{2}\right)$, along with the following probability density function:

$$\begin{array}{cc} p\left(u_{mi}|{\vec{Z}}_{m},\Theta \right)=\; & \frac{\exp\left\{\left(\tau \mu^{T}+\frac{1}{\delta }z_{mi}^{T}\right)u_{mi}-\frac{1}{2\delta }u_{mi}^{T}u_{mi}\right\}}{\zeta \left(\tau \mu +\frac{1}{\delta }z_{mi},\;\frac{1}{2\delta }I_{2}\right)}. \end{array}$$

## Appendix D. Comparison of Latent Variable Generation Methods in Step 2

In this section, we generate ten latent variable samples ${\vec{U}}_{m}$ with $n_{m}=50$ using the true parameters $\Theta_{0}$ and the true latent variable ${\vec{Z}}_{m0}$. Figure A1 illustrates the samples generated by the GA and FB methods, respectively. The results indicate that the data produced by the GA method effectively captures the trend of the true values, yielding an RMSE of 0.130 and a Pearson correlation coefficient of 0.98. The FB distribution method is more accurate, achieving a lower variance with an RMSE of 0.106 and a Pearson correlation of 0.99.

Additionally, we compare the computation time required for these two methods in sampling ${\vec{U}}_{m}$ across varying numbers of data points $n_{m}$ for *m*-th batch. The computation time for each method is averaged over 100 replications. The results, presented in Table A1, demonstrate that the GA method is much more time-efficient than the FB method.

**Table A1:** Computation time (in seconds) for the GA method and the FB method in sampling ${\vec{U}}_{m}$ across different numbers of data points, based on 100 replications.

	$n_{m}=10$	$n_{m}=30$	$n_{m}=60$
GA	0.0012	0.0023	0.0056
FB	0.0045	0.0190	0.0949

## Appendix E. Proof for Hyperparameter Consistency

We consider the estimation of the hyperparameter $\theta_{K}$ and prove that the estimator obtained by maximizing the marginal likelihood is consistent when the number of data points for each curve is sufficiently large, as established by [43]. The marginal log-likelihood is given by

$$\begin{array}{c} \ell \left(\theta_{K}\right)=\sum_{m=1}^{M}\log\int \exp\left\{\log p\left({\vec{Y}}_{m}|{\vec{Z}}_{m}\right)+\log p\left({\vec{Z}}_{m}\right)\right\}d{\vec{Z}}_{m}. \end{array}$$

Define $\Psi \left({\vec{Z}}_{m}\right)=\log p\left({\vec{Y}}_{m}|{\vec{Z}}_{m}\right)+\log p\left({\vec{Z}}_{m}\right)$, with ${\hat{\vec{Z}}}_{m}$ being the maximizer of $\Psi \left({\vec{Z}}_{m}\right)$. Consequently, the Laplace approximation for $\ell (\theta_{K})$ can be expressed as

$$\begin{array}{c} \ell_{\mathrm{appr}.}\left(\theta_{K}\right)=\sum_{m=1}^{M}\Psi \left({\hat{\vec{Z}}}_{m}\right)+nM\log\left(2\pi \right)-\frac{1}{2}\sum_{m=1}^{M}\log\left|\frac{1}{\sigma_{\epsilon }^{2}}A^{2}⊗I_{n}+{\left[I_{2}⊗K_{m}\right]}^{-1}\right|. \end{array}$$

Let ${\hat{\theta }}_{K}$ denote the maximum likelihood estimator based on this approximation, such that ${\left.\frac{\partial \ell_{\mathrm{appr}.}\left(\theta_{K}\right)}{\partial \theta_{K}}\right|}_{\theta_{K}={\hat{\theta }}_{K}}=0$. Under the usual regularity conditions on $\ell (\theta_{K})$, and assuming ${\hat{\theta }}_{K}$ is an interior point in a neighborhood of the true value ${\theta_{K}}_{0}$, we can apply a Taylor expansion around ${\theta_{K}}_{0}$:

$$\begin{array}{c} {\left.\frac{1}{M}\frac{\partial \ell (\theta_{K})}{\partial \theta_{K}}\right|}_{\theta_{K}={\hat{\theta }}_{K}}={\left.\frac{1}{M}\frac{\partial \ell (\theta_{K})}{\partial \theta_{K}}\right|}_{\theta_{K}={\theta_{K}}_{0}}+{\left.\frac{1}{M}\frac{\partial^{2}\ell \left(\theta_{K}\right)}{\partial \theta_{K}\partial {\theta_{K}}^{T}}\right|}_{\theta_{K}={\theta_{K}}_{0}}\left({\hat{\theta }}_{K}-{\theta_{K}}_{0}\right)+O_{p}\left(1\right)\left(∥{\hat{\theta }}_{K}-{\theta_{K}}_{0}{∥}^{2}\right). \end{array}$$

Given sufficient regularity conditions, we have

$$\begin{array}{cc} {\left.\frac{1}{M}\frac{\partial^{2}\ell \left(\theta_{K}\right)}{\partial \theta_{K}\partial {\theta_{K}}^{T}}\right|}_{\theta_{K}={\theta_{K}}_{0}} & =O_{p}\left(1\right),\;{\left.\frac{1}{M}\frac{\partial \ell (\theta_{K})}{\partial \theta_{K}}\right|}_{\theta_{K}={\theta_{K}}_{0}}=O_{p}(M^{-\frac{1}{2}}),\\ {\left.\frac{1}{M}\frac{\partial \ell (\theta_{K})}{\partial \theta_{K}}\right|}_{\theta_{K}={\hat{\theta }}_{K}} & ={\left.\frac{1}{M}\frac{\partial \ell_{\mathrm{appr}.}\left(\theta_{K}\right)}{\partial \theta_{K}}\right|}_{\theta_{K}={\hat{\theta }}_{K}}+O\left(nM^{-2}\right) \end{array}$$

It follows that

$$\begin{array}{cc} {\hat{\theta }}_{K}-{\theta_{K}}_{0} & =\frac{{\left.\frac{1}{M}\frac{\partial \ell (\theta_{K})}{\partial \theta_{K}}\right|}_{\theta_{K}={\hat{\theta }}_{K}}-{\left.\frac{1}{M}\frac{\partial \ell (\theta_{K})}{\partial \theta_{K}}\right|}_{\theta_{K}={\theta_{K}}_{0}}}{O_{p}\left(1\right)}\\ \; & ={\left.\frac{1}{M}\frac{\partial \ell_{\mathrm{appr}.}\left(\theta_{K}\right)}{\partial \theta_{K}}\right|}_{\theta_{K}={\hat{\theta }}_{K}}+O\left(nM^{-2}\right)+O_{p}(M^{-\frac{1}{2}})\\ \; & =O_{p}(\max\{nM^{-2},M^{-\frac{1}{2}}\}). \end{array}$$

The estimator ${\hat{\theta }}_{K}\to {\theta_{K}}_{0}$ almost surely as *M* tends to infinity and $n=o(M^{2})$ under certain regularity conditions.

## Appendix F. Simulation for *τ* Effect

In this simulation, which shares the setting of Simulation 1 with n=30 and M=30, we investigate the impact of the parameter τ on other parameters. As the true value of τ, denoted as $\tau_{0}$, increases, it indicates a higher concentration of observed data within a specific segment of the ellipse, which subsequently affects the bias of the semi-major and semi-minor axes, the center position, and the estimation of τ. According to Figure A2, when $\tau_{0}$ is less than 2, the biases in these parameters are relatively small. However, as $\tau_{0}$ becomes larger, the biases increase. This phenomenon occurs because excessive data concentration leads to initial estimates that deviate significantly from the true parameter values, making it challenging to achieve accurate results through iterative processes.

## Appendix G. Evaluation on the Convergence of MCMC Chains

To evaluate the convergence of the MCMC chains, We select small values for both the bath number and the number of data points per bath, specifically using M,n=10. The observation points are concentrated in a specific region of the elliptical curve with τ=1. In this context, simulated data are generated, producing five parallel chains of length 10,000 using the GA method to sample $\vec{U}$. Following the guidelines in [33,74], we employ a cutoff value of 1.1 to determine convergence. Figure A3 illustrates the median PSRF and its 97.5% quantile for each parameter. Convergence is deemed satisfactory when the number of iterations s≥5000.
